# Meta-analyses Using Individual Participant Data From Cardiovascular Cohort Studies in Japan: Current Status and Future Directions

**DOI:** 10.2188/jea.JE20130177

**Published:** 2014-03-05

**Authors:** Yoshitaka Murakami

**Affiliations:** Department of Medical Statistics, Shiga University of Medical Science, Otsu, Japan; 滋賀医科大学医療統計学部門

**Keywords:** meta-analysis, individual participant data, premature death, cardiovascular disease, population attributable fraction

## Abstract

Meta-analysis of individual participant data (IPD meta-analysis) has several advantages over meta-analysis using aggregated published data, including the possibility of using statistical methods such as a fine stratification analysis, interaction analysis between 2 risk factors, and absolute risk estimation. The Evidence for Cardiovascular Prevention from Observational Cohorts in Japan Study (EPOCH-JAPAN), which was initiated in 2005, is a collaborative research project for IPD meta-analysis and includes 13 participating cohort studies in Japan. We generated 2 pooled databases with data on all-cause mortality (*n* = 199 047) and cardiovascular outcomes (*n* = 90 528) and applied a stratified Cox model to account for the different baseline hazards between cohorts. The results of our analyses show the age- and sex-specific associations between all-cause and cardiovascular disease mortality and established cardiovascular risk factors (blood pressure, smoking, total cholesterol, proteinuria, and kidney function). During the 9 years of its existence, the results generated by EPOCH-JAPAN have had important implications for clinical medicine and public health policy in Japan. The project is expected to draw upon new analytical methods such as interaction analysis and absolute risk evaluation in the near future. We believe that, over the next decade, this project will continue to provide new insights that can be applied to research on other Asian populations.

## Abstract (ja)

個人単位のデータによるメタアナリシス（IPD meta-analysis）は出版されたデータを用いたメタアナリシスに比べ、詳細な層別解析、2つの危険因子間の交互作用解析、絶対リスクの推定など様々な統計手法を利用できる可能性をもっている。EPOCH-JAPAN (The Evidence for Cardiovascular Prevention from Observational Cohorts in Japan Study)は2005年に開始されたIPD meta-analysis の共同研究プロジェクトであり、日本の13つのコホート研究が参加している。全死亡をエンドポイントとするデータベース（19,9047人）と循環器疾患死亡をエンドポイントとするデータベース（90,528人）の2つの統合データベースを生成し、コホート間のベースラインハザードの違いを考慮した層別Cox回帰モデルを適用した。私たちの分析結果より、全死亡および循環器死亡と循環器疾患における確立された危険因子(血圧、喫煙、総コレステロール、蛋白尿、腎機能)との関連が示された。9年の研究期間を経てEPOCH-JAPANから生み出された成果は、わが国の臨床医学、公衆衛生政策の両面において重要な貢献を果たしてきた。相互作用解析や絶対リスク評価などの新しい解析方法における将来的な進展が本プロジェクトから期待される。本プロジェクトから、他のアジア諸国の集団に対する研究に応用できる視点を、今後10年継続して提供することを確信する。

## INTRODUCTION

**Figure 1.**
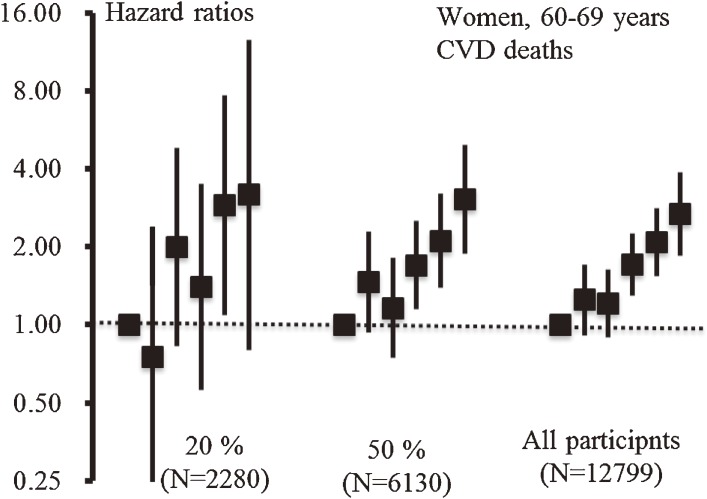
Relationship between blood pressure and cardiovascular disease deaths among different sampling sets: women aged 60–69 years, EPOCH-JAPAN. Three different sampling sets were generated and analyzed separately. The samples were set as follows: 20% sampling (2280 participants), 50% sampling (6130 participants), and all participants (12 799). Blood pressure measurements (unit: mm Hg; SBP, systolic blood pressure; DBP, diastolic blood pressure) were grouped into 6 categories (SBP < 120 and DBP < 80 (reference), 120 ≤ SBP < 130 or 80 ≤ DBP < 85, 130 ≤ SBP < 140 or 85 ≤ DBP < 90, 140 ≤ SBP < 160 or 90 ≤ DBP < 100, 160 ≤ SBP < 180 or 100 ≤ DBP < 110, and 180 ≤ SBP or 110 ≤ DBP). The error bars show 95% CIs.

The proliferation of information technology during the past 2 decades has given researchers unprecedented access to a wider range of scientific literature. This has created new opportunities to conduct meta-analyses, ie, systematic overviews of the literature using quantitative methods that allow researchers to enhance the accuracy of their findings by combining information from multiple studies.^1^ Individual participant data (IPD) meta-analysis (sometimes referred to as individual participant data meta-analysis or pooled analysis) has several advantages over meta-analysis using aggregated published data.^2^^,^^3^ One advantage of IPD meta-analyses using individual-based datasets is that they can make use of statistical techniques such as a fine stratification analysis, interaction analysis between 2 risk factors, and absolute risk estimation. None of these methods can be applied to a single-site cohort study because sample size is usually insufficient. Figure 1 shows the relationship between blood pressure (BP) category and cardiovascular disease (CVD) mortality in 3 different sampling sets generated by random sampling from the pooled database. We found that the confidence intervals for the relationship between systolic BP and CVD mortality narrowed as the sample size increased. Moreover, these results show that an apparent trend with narrow confidence intervals can only be achieved when the sample size is sufficient.

The Evidence for Cardiovascular Prevention from Observational Cohorts in Japan Study (EPOCH-JAPAN), which was initiated in 2005, is a collaborative research project for IPD meta-analyses of CVD in Japan. This article will outline the design, methods, and main results of the EPOCH-JAPAN study. Finally, I will offer my perspective on the future of the project and its implications for future research.

## BRIEF INTRODUCTION OF EPOCH-JAPAN

### The collection of cohort studies

**Figure 2.**
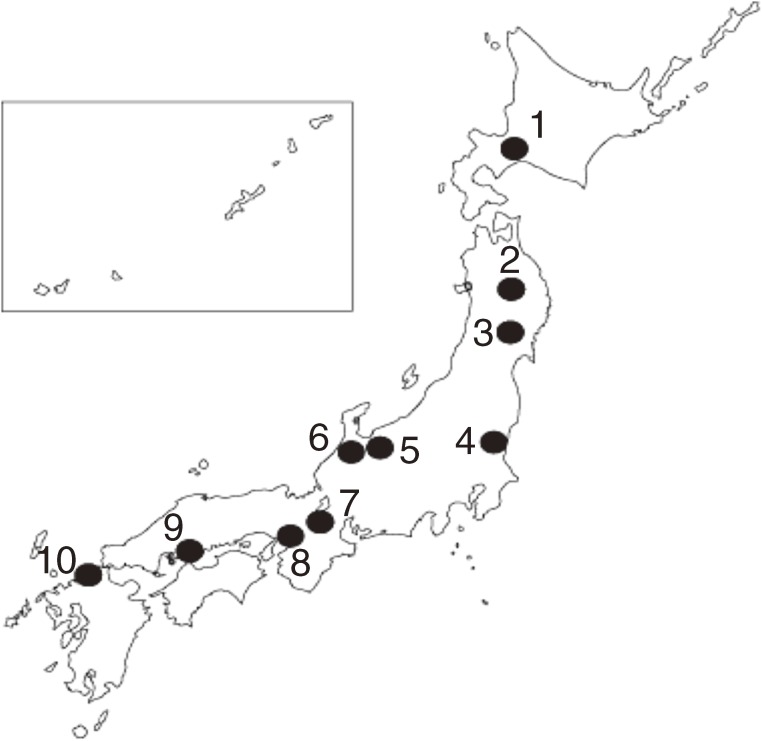
The EPOCH-JAPAN cohort study sites. Each point on the map represents a study site where an EPOCH-JAPAN cohorts were located. The names of the studies are as follows: 1: Tanno-Sobetsu, 2: Ohasama, 3: Osaki, 4: Ibaraki prefecture, 5: YKK workers, 6: Oyabe, 7: Shiga Health Insurance, 8: Suita, 9: Radiation Effects Research Foundation, 10: Hisayama. Three nationwide cohort studies (JACC study, NIPPON DATA80, and NIPPON DATA90) were also included in EPOCH-JAPAN.

EPOCH-JAPAN comprises 13 cohort studies of the relationships between health measures (including laboratory measures and survey items on lifestyle and behavioral factors) and disease outcomes (total and disease-specific mortality) in the Japanese population. Figure 2 shows the locations of these cohorts, which include 10 single-site and 3 nationwide cohort studies distributed throughout Japan. Only those cohorts that collected results from health examinations, had a mean follow-up period of approximately 10 years, and included more than 1000 participants were eligible for inclusion in EPOCH-JAPAN.

We then generated 2 pooled databases for different outcome measures. While the first contained data from 199 047 participants showing all-cause deaths only, the second included data on cardiovascular outcomes from 90 528 participants. In some analyses, we included follow-up data only from participants aged 40 to 90 years. This was considered appropriate because the end-point of follow-up varied across cohorts. A full list of the members of the EPOCH-JAPAN Study Research Group is shown in the Appendix.^4^

### Statistical analyses

The stratified Cox proportional hazards model, which can account for differences in baseline hazards between strata, was the default method used for most analyses in EPOCH-JAPAN. Because the baseline hazard (absolute mortality rate) of each cohort was likely to be different, the stratified Cox model was considered one of the best models to satisfy this assumption. This method can be easily applied using standard statistical software such as SAS, STATA, and SPSS and has been widely used in other IPD meta-analyses.^3^ In addition, we used Poisson regression to estimate absolute risk (ie, mortality rate). In our Poisson regression analyses, we modeled a separate indicator (dummy) variable for each cohort to adjust for differences in baseline mortality rates between cohorts.

The age- and sex-specific results generated have given us key insights into various exposure–disease relationships in different groups within the Japanese population. For the purposes of analysis, participants in the all-cause mortality database were stratified into 10-year age groups, from 40 to 80 years, which were analyzed using separate models. Population attributable fraction (PAF), which shows the impact of an exposure across the population as a whole, was also an important outcome measure in EPOCH-JAPAN. Most articles published from EPOCH-JAPAN have used this measure to represent the potential impact of specific risk factors on the Japanese population.

In our IPD meta-analyses, correction for regression dilution bias was sometimes done using the MacMahon and Peto method. This bias correction was applied in our investigation of the relationship between BP and all-cause mortality.^4^ All statistical analyses were performed using SAS 9.13 (SAS Institute Inc).

## RESULTS

Several reports have already been published from EPOCH-JAPAN.^4^^–^^9^ Brief summaries of each study are shown below.

### BP and all-cause mortality^4^

**Figure 3.**
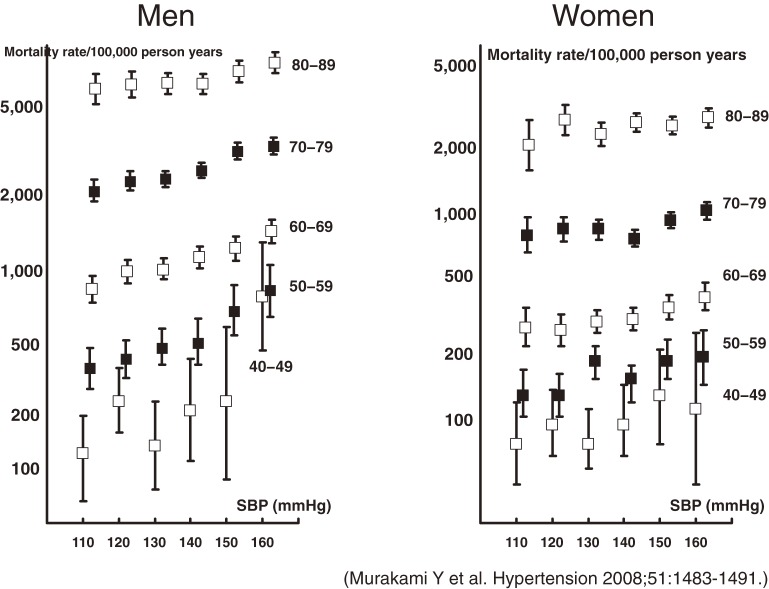
Relationship between systolic blood pressure and adjusted mortality rate by age group. Each square represents a multivariate estimate of mortality rates after adjusting for smoking status, alcohol use, and body mass index; the error bars show 95% CIs. The number of events in each age category are as follows: men aged 40–49 years: 137; age 50–59 years: 566; age 60–69 years: 1900; age 70–79 years: 3782; age 80–89 years: 2183; women aged 40–49 years: 128; age 50–59 years: 518; age 60–69 years: 1392; age 70–79 years: 2708; age 80–89 years: 2258.

Hypertension is a leading cause of premature death, and cardiovascular disease is a major contributor to total mortality. To examine possible ways to reduce avoidable deaths from hypertension, we examined 13 cohorts from EPOCH-JAPAN to assess the impact of hypertension on total mortality in Japan. The results of our multivariate model (Figure 3) show the relationship between BP and mortality rate by age group. While adjusted mortality rates rose as BP increased, this effect was most pronounced in younger men and women. The PAF for hypertension was approximately 20% when only participants with normal BP were included in the reference group and 10% when we included those with prehypertension in the reference group. These results indicate that high BP increased the risk of total mortality and that this effect was strongest among younger Japanese.

### Smoking and all-cause mortality^5^

**Figure 4.**
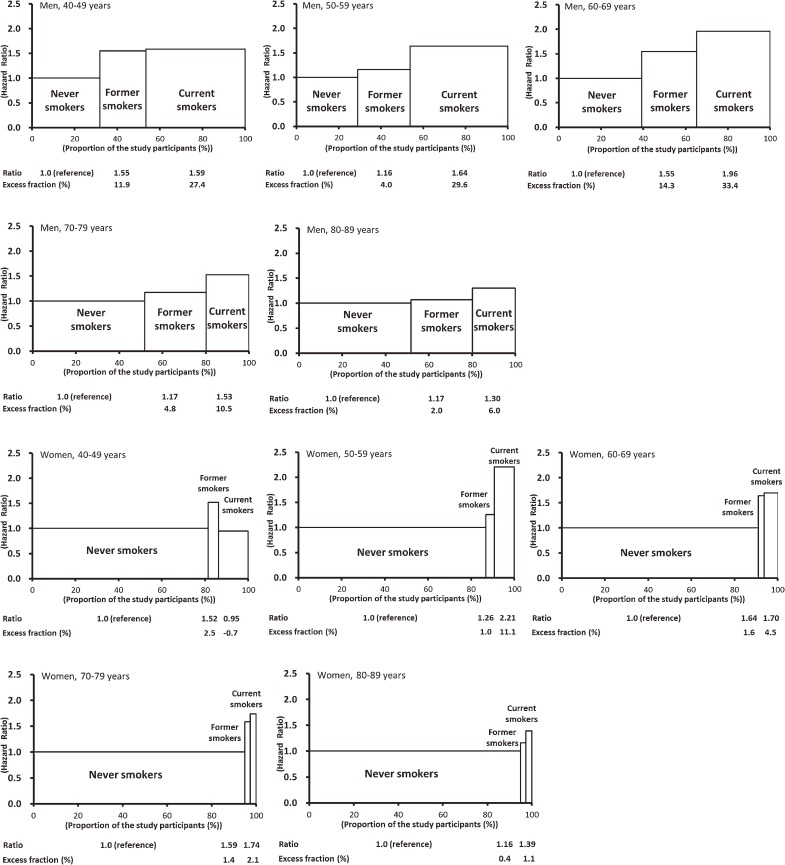
Age- and sex-specific hazard ratios and their population attributable fractions in EPOCH-JAPAN. In each graph, the X-axis represents the proportion of the study population in each smoking status category (never smoker, former smoker, and current smoker). The Y-axis shows the hazard ratios for each category of smoking status. The excess fraction shows the proportion of excess deaths in each smoking status category as compared with the reference group (never smokers).

To determine age- and sex-specific PAFs and the number of premature deaths attributable to smoking in an Asian population, we examined hazard ratios and corresponding PAFs using EPOCH-JAPAN. Figure 4 shows the age-specific hazard ratios according to smoking status and the PAFs from EPOCH-JAPAN. The overall proportion of deaths attributable to smoking was 24.6% in men and 6.0% in women. The age-specific PAF was highest in men aged 60 to 69 years (47.7%) and in women aged 50 to 59 years (12.2%). Among those aged 70 to 79 years and 80 to 89 years, the PAFs were 15.4% and 8.0%, respectively, in men and 3.5% and 1.5% in women. Our results show that age-specific PAFs for Japanese men were much larger than those reported in studies of other Asian countries. The corresponding total number of premature deaths annually due to smoking in Japan, as calculated from our PAFs, was 121 854 (109 998 men; 11 856 women).

### Cardiovascular disease mortality and its established risk factors

#### Blood pressure^6^

The risk of CVD according to BP category and age group has not been thoroughly investigated in an Asian population. To investigate the risk of CVD mortality according to BP category and age group, we examined individual data from 10 cohorts, including 67 309 individuals aged 40 to 89 years who had not received a diagnosis of CVD at baseline. We observed an elevated risk of CVD in very old participants (age, 75–89 years) with a systolic/diastolic blood pressure (SBP/DBP) of 130/85 mm Hg or higher and a significant increase among members of other age groups (65–74 years and 75–89 years) with an SBP/DBP of 120/80 mm Hg or higher. The PAFs for CVD mortality ranged from 23.4% to 60.3% among very old and middle-aged participants, respectively, with an SBP/DBP lower than 120/80 mm Hg. These PAFs suggest that maintaining a low BP is an important strategy for primary CVD prevention, even in elderly populations.

#### Smoking^7^

To inform effective smoking prevention strategies that safeguard the nation’s health, large-scale analyses using pooled data are needed in order to provide reliable information on the adverse effects of smoking and its impact on mortality, particularly among individuals with hypertension or high serum cholesterol. In our multivariate model, adjusted hazard ratios in male and female current smokers with hypertension were 2.57 (95% CI: 1.51–4.38) and 6.14 (95% CI: 3.49–10.79) for coronary heart disease, respectively, and 3.28 (95% CI: 1.89–5.71) and 1.61 (95% CI: 0.81–3.18) for cerebral infarction, as compared with participants with neither risk factor. The percentages of deaths attributable to coexistence of current smoking and hypertension in men and women were 24.6% and 9.6%, respectively, for coronary heart disease and 28.1% and 2.0% for cerebral infarction. Smokers with high serum cholesterol were broadly comparable to hypertensive smokers only with respect to coronary mortality risk: the hazard ratios were 4.19 (95% CI: 2.33–7.53) for men and 3.90 (95% CI: 1.57–9.67) for women when compared with participants with neither risk factor. Particular attention should be given to smokers with hypertension or high serum cholesterol, due to their high risk of mortality from cardiovascular disease.

#### Total cholesterol^8^

We examined a total of 65 594 participants aged 40 to 89 years without a history of cardiovascular disease, which we divided into 2 age groups: middle-aged (40–69 years; mean age, 55 years) and elderly (70–89 years; mean age, 75 years). In our multivariate model, the adjusted hazard ratios for coronary heart disease among men in the highest total cholesterol (TC) category (≥6.21 mmol/L) as compared with the lowest category (<4.14 mmol/L) were 2.52 (95% CI: 1.15–5.07) for middle-aged participants and 2.77 (95% CI: 1.09–7.03) for elderly participants. In women, the hazard ratios for the highest TC category (≥6.72 mmol/L) as compared with the lowest category (<4.66 mmol/L) were 3.20 (95% CI: 1.44–7.09) for middle-aged participants and 1.02 (95% CI: 0.42–2.49) for elderly participants. Although high serum TC levels were associated with coronary heart disease in middle-aged Japanese men and women, evidence of an association in elderly Japanese individuals remains limited.

#### Proteinuria and reduced kidney function^9^

Few studies have examined whether proteinuria and estimated glomerular filtration rate (eGFR) are independently associated with cardiovascular disease in an Asian population. Using data from 7 prospective cohorts in EPOCH-JAPAN, we investigated the influence of proteinuria (≥1+ on dipstick) and reduced eGFR on the risk of cardiovascular disease mortality in 39 405 participants (40–89 years) without kidney failure. Proteinuria was associated with 1.75-fold (95% CI: 1.44–2.11) increased risk of cardiovascular disease mortality, after adjustment for potential confounding factors. Additionally, in our multivariate model, we found a negative linear association between cardiovascular disease mortality and low eGFR (*P*_trend_ < 0.001). The hazard ratio for subjects with an eGFR of less than 45 mL/minute/1.73 m^2^ was 2.22 (95% CI: 1.60–3.07), when compared with those with an eGFR of 90 mL/minute/1.73 m^2^ or greater. The hazard ratio for participants with both proteinuria and an eGFR of less than 45 mL/minute/1.73 m^2^ was 4.05 (95% CI: 2.55–6.43) when compared with subjects with neither of these risk factors. These results suggest that proteinuria and a low eGFR are independent risk factors for cardiovascular disease mortality in the Japanese population.

## DISCUSSION AND FUTURE DIRECTIONS

We have described our data-pooling project for cardiovascular epidemiology in Japan, EPOCH-JAPAN, and have presented some of the results it has generated to date. This project is ongoing and is being constantly updated and expanded by recruiting other cohort studies. The study collaborators are constantly encouraged to conduct new analyses, and, in particular, to undertake interaction analyses^10^^,^^11^ and evaluations of absolute risk.^4^ In these analyses, we tested for interaction, or the effect when 2 risk factors co-occur, using a likelihood ratio test to compare goodness-of-fit of the main model against that of the alternative model including the interaction terms. In most cases, this type of interaction analysis requires a large sample size because the inclusion of interaction terms increases the number of model parameters. This approach is only possible when a large database of participants is available. The most significant advantage of this approach for interaction analyses is that it allows us to draw comprehensive conclusions regarding the presence of interaction effects.^10^^,^^11^

When we classified our study population according to level of exposure, the number of cases in the exposed group would often be too small to yield reliable estimates of disease risk. For example, because of the difficulty in recruiting a sufficient sample of people with a rare cardiovascular risk factor such as isolated systolic hypertension, its impact on mortality could not be investigated using a single-site cohort study. While there had previously been much uncertainty regarding the association between isolated diastolic hypertension and cardiovascular disease, an IPD meta-analysis from the Asia-Pacific region produced new insights by examining this issue using large datasets.^12^ While small exposure groups and low numbers of cases have been limiting factors in a number of epidemiologic studies, particularly those of rare diseases, we have shown that IPD meta-analyses can provide an effective approach for studying a range of epidemiologic topics.

Nearly a decade has passed since we initiated this IPD meta-analysis project. During this period, several of its research outputs have been used to provide important evidence for drafting clinical guidelines and informing government health policy in Japan. As rapid economic growth in Asia accelerates lifestyle changes in many populations, the burden of chronic disease is likely to increase in a number of countries during the next decade. Over the coming years, we believe that our research will continue to provide further insights that can be applied to research on other Asian populations as they undergo epidemiologic transition.

## ONLINE ONLY MATERIALS

Abstract in Japanese.
