# Supplementary material for: Meta-analyses Using Individual Participant Data From Cardiovascular Cohort Studies in Japan: Current Status and Future Directions
Source: J Epidemiol. 2014 Mar 5;24(2):96–101. doi: 10.2188/jea.JE20130177 (PMC3956690; doi:10.2188/jea.JE20130177)
Supplement: Abstract in Japanese. [file je-24-096-s001.pdf]

日本の循環器疫学コホート研究を対象とした個人単位データを用いたメタアナリシス:現状と将来展望

村上義孝
滋賀医科大学医療統計学部門

個人単位のデータによるメタアナリシス（IPD meta-analysis）は出版されたデータを用いたメタアナリシスに比べ、詳細な層別解析、2つの危険因子間の交互作用解析、絶対リスクの推定など様々な統計手法を利用できる可能性をもっている。EPOCH-JAPAN（The Evidence for Cardiovascular Prevention from Observational Cohorts in Japan Study)は2005年に開始されたIPD meta-analysis の共同研究プロジェクトであり、日本の13つのコホート研究が参加している。全死亡をエンドポイントとするデータベース（19,9047人）と循環器疾患死亡をエンドポイントとするデータベース（90,528人）の2つの統合データベースを生成し、コホート間のベースラインハザードの違いを考慮した層別Cox回帰モデルを適用した。私たちの分析結果より、全死亡および循環器死亡と循環器疾患における確立された危険因子(血圧、喫煙、総コレステロール、蛋白尿、腎機能)との関連が示された。9年の研究期間を経てEPOCH-JAPANから生み出された成果は、わが国の臨床医学、公衆衛生政策の両面において重要な貢献を果たしてきた。相互作用解析や絶対リスク評価などの新しい解析方法における将来的な進展が本プロジェクトから期待される。本プロジェクトから、他のアジア諸国の集団に対する研究に応用できる視点を、今後10年継続して提供することを確信する。

キーワード:メタアナリシス、個人単位データ、早世死亡、循環器疾患、集団寄与危険割合
